# Aptamer-Mediated Targeted Delivery of Therapeutics: An Update

**DOI:** 10.3390/ph9040069

**Published:** 2016-11-03

**Authors:** Silvia Catuogno, Carla L. Esposito, Vittorio de Franciscis

**Affiliations:** Istituto per I’Endocrinologia e I’Oncologia Sperimentale del CNR “G. Salvatore”, Via S. Pansini 5, 80131 Naples, Italy; silviacatuogno@virgilio.it (S.C.); c.esposito@mail.ieos.cnr.it (C.L.E.)

**Keywords:** aptamers, drug delivery, targeted therapy

## Abstract

The selective delivery of drugs in a cell- or tissue-specific manner represents the main challenge for medical research; in order to reduce the occurrence of unwanted off-target effects. In this regard, nucleic acid aptamers have emerged as an attractive class of carrier molecules due to their ability to bind with high affinity to specific ligands; their high chemical flexibility; as well as tissue penetration capability. To date, different aptamer-drug systems and aptamer–nanoparticles systems, in which nanoparticles function together with aptamers for the targeted delivery, have been successfully developed for a wide range of therapeutics, including toxins; peptides; chemotherapeutics and oligonucleotides. Therefore, aptamer-mediated drug delivery represents a powerful tool for the safe and effective treatment of different human pathologies, including cancer; neurological diseases; immunological diseases and so on. In this review, we will summarize recent progress in the field of aptamer-mediated drug delivery and we will discuss the advantages, the achieved objectives and the challenges to be still addressed in the near future, in order to improve the effectiveness of therapies.

## 1. Introduction

Most of therapeutic agents approved by the Food and Drug Administration (FDA) for the treatment of different human pathologies show no selectivity for diseased cells, thus reducing their therapeutic index and producing significant toxicity and side effects associated to the non-specific bio-distribution in the body. Therefore, the research of strategies for therapeutic targeted delivery has become one of the most important challenges field. The basic idea is to use targeting ligands that specifically recognize diseased cells and are able to act as carriers for therapeutics, in order to improve the efficacy and the tolerability of the treatment.

**Figure 1:**
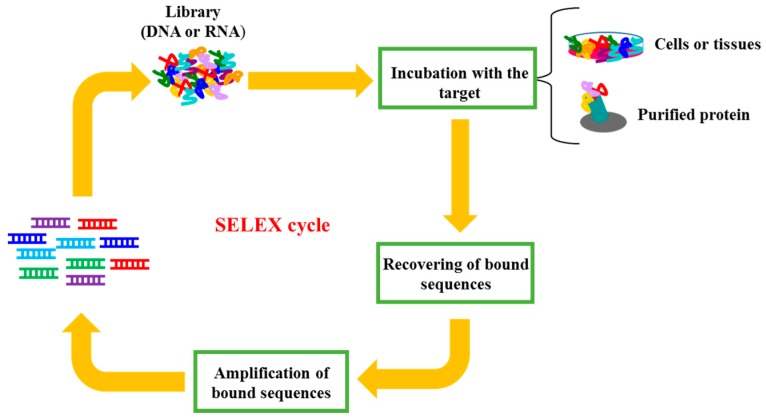
Schematic representation of SELEX (Systematic Evolution of Ligand by EXponential enrichment). SELEX process involves repeated steps of: (i) incubation of a high complexity library with a target of interest; (ii) recovery of bound sequences and (iii) amplification of bound sequences.

In this regard, the development of the SELEX (Systematic Evolution of Ligand by EXponential enrichment) technology have raised interesting targeting molecules, called aptamers. Aptamers are single stranded DNA or RNA oligonucleotides that bind with high affinity to specific target molecules by folding into unique three-dimensional structures. The process of aptamer selection involves repeated steps of incubation of a high complexity library with a target of interest (Figure 1), which may vary from purified proteins to complex targets, such as cells or tissues.

So far, several variants of SELEX protocol have been successfully applied to the isolation of a wide range of aptamers against therapeutically relevant targets [1,2]. In most cases, aptamers provide their therapeutic effect by inhibiting their proper target and thus attenuating the corresponding downstream molecular pathway. Among them, aptamers directed against cell surface epitopes represent a very attractive chance for therapeutic targeted delivery. Indeed, once bound to cell surface receptors, some aptamers show the ability to internalize in a target-mediated manner and may be used to drive a secondary reagent exclusively to the cell population over-expressing the aptamer target. Even if the specific mechanisms responsible for this internalization process are still not completely understood, a growing number of targeted delivery strategies using cell-specific aptamers have been proposed [3]. In addition to their rapid internalization rate into diseased cells, aptamers possess many advantages over monoclonal antibodies and peptides. As compared to monoclonal antibodies, to which aptamers are often likened, they have comparable affinity (Kd in the low nano or picomolar range) and specificity for their ligands and show easier production, cost-effectiveness and batch-to-batch fidelity. In addition, aptamers are poorly or not immunogenic and small in size [4]. Research on aptamer chemistry has progressed and to date several nucleotide and backbone modifications have been proposed to increase nucleobase diversity and enhance aptamer serum stability and pharmacokinetic [5,6]. Moreover, chemical modifications to conjugate them with secondary reagents, including imaging probe, drugs or nanoparticles (NPs) have been proposed [7,8].

Aptamer-based delivery strategies are mainly based on two different approaches: (1) Aptamer-secondary reagent direct conjugation and (2) Aptamer-functionalization of nanomaterials. In both cases, the aptamer is used as a targeting moiety to recognize diseased tissues and to specifically attack them with the therapeutic compounds. So far, aptamers have been covalently or physically functionalized with therapeutic compounds including chemotherapeutics, toxins, and therapeutic oligonucleotides (siRNAs, miRNAs or antimiRs) and many methods of aptamer functionalization have been proposed as valid means to improve the specificity of nanoparticles. These approaches have been applied for the specific targeted delivery in different human diseases, including cancer [9] and immunological disorders [10].

As witnessed by recent reviews discussing multiple specific aspects [11,12], the interest in aptamers as highly selective and safe delivery carriers is rapidly increasing. In this review, we will provide a comprehensive overview of the most interesting examples and we will report the most recent advances, underlining the conjugation strategies used and the achieved results. Current challenges and future perspectives for an aptamer-mediated cell-specific delivery will be discussed.

## 2. Aptamer-Drug and Oligonucleotide Systems

Taking advantage of aptamer chemical features, several strategies in which aptamers have been directly conjugated to different secondary reagents, such as small drugs or therapeutic oligonucleotides (siRNAs, miRNAs, antimiRs), have been proposed as simple and cost effective means to selectively drive them to target cells.

### 2.1. Aptamer-Drug Conjugates

For targeted drug therapy, drug molecules may be simply attached to aptamers by covalent or noncovalent conjugation. The majority of published studies refers to the conjugation of chemotherapeutic agents to tumor-targeting aptamers and, in this regard, one of the more used drugs is Doxorubicin (DOX). This molecule is an anthracycline-based chemotherapeutic that disrupts replication and transcription, inducing cancer cell death through the intercalation into DNA, preferentially occurring in regions rich in cytosine (C) and guanine (G) and inhibiting the DNA topoisomerase II. Despite Doxorubicin, being widely used for the treatment of several type of cancers, causes a dose-dependent cardiotoxicity that greatly limits its efficacy [13].

**Figure 2:**
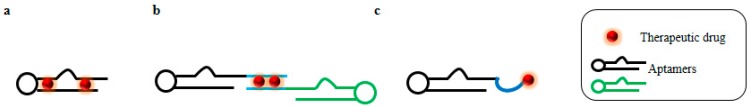
Schematic representation of aptamer-drug conjugates. (**a**,**b**) Examples of non-covalent aptamer-drug conjugation through intercalation; (**c**) Scheme of an aptamer-drug covalent conjugation.

Based on the DOX mechanism of action, the ability to transport it via its direct intercalation into aptamers, with the advantage of having a very simple conjugation strategy that does not require any chemical modification, has been explored. In the last decade, this strategy has been used to generate DOX-physical conjugates (Figure 2a) with several targeting aptamers such as the anti- prostate-specific membrane antigen (PSMA) A10 [14] and the anti-epithelial cell adhesion molecule (EpCAM) EpDT3 [15] RNA aptamers or the anti- protein tyrosine kinase (PTK)-7 sgc8 [16], the anti-Mucin (MUC)1 [17] and the anti-HER-2 [18] DNA aptamers. All these studies showed a good cellular uptake and drug release in aptamer target positive cells and a reduced cytotoxicity of aptamer-drug complexes in negative cells as compared to Doxorubicin alone in vitro. These data indicate that because of the aptamer conjugation there is a decreased unspecific uptake allowing a reduction of the overall toxicity of DOX. The original strategy has been also further optimized to improve DOX intercalation efficiency or complex recognition ability. By using a human hepatocellular carcinoma cell line specific aptamer (TLS11a) modified with a long Guanine-Cytosine tail, Meng et al. [19] achieved a higher Doxorubicin-aptamer ratio, consequently increasing the killing efficiency of target cancer cells both in vitro and in vivo. Hepatocellular carcinoma targeting was also achieved by generating a Drug-DNA Adduct (DDA) containing DOX and the anti nucleolin aptamer AS1411 [20].

More recently, a similar approach has been used with an anti CD-38 aptamer by Wen et al. [21] to generate a novel aptamer-drug conjugate for multiple myeloma targeting. In order to enhance DOX loading, the aptamer was synthesized with CG-cargo achieving a molecule able to incorporate five DOX payloads. The study demonstrated that the generated complex was able to specifically bind the target cells and upon internalization released the drug through a pH-controlled mechanism induced by a structural change. Importantly, the treatment with the conjugate specifically induced apoptosis, reduced colony formation in vitro, and inhibited tumor growth in mice bearing orthotopic multiple myeloma tumors.

A broadened recognition capability of aptamer-drug systems has instead been obtained by Zhu et al. [22] through the development of a DOX-bispecific aptamer complex (Figure 2b). They generated a bi-specific drug carrier (SD) by conjugating sgc8c and sgd5a monovalent aptamers, recognizing T lymphoblast CEM and B lymphocyte Toledo cells, respectively [23,24]. The two aptamers were connected through a dsDNA linker containing multiple DOX intercalation sites and the resulting SD-DOX conjugate showed bispecific target cell binding, drug delivery and cytotoxicity.

Further, a tridentate anti-MUC-1 DNA aptamer, containing three repeats of the active targeting region of the oligonucleotide, was designed enhancing both DOX intercalation and selective cytotoxicity [25].

In addition to Doxorubicin, a direct aptamer-drug physical conjugation has been applied for the interaction between a G-quadruplex structure aptamer (the anti-nucleolin AS1411 DNA aptamer) and a photodynamic agent (TMPyP4) [26]. Based on TMPyP4 aromatic and cationic properties, in the complex six molecules of this agent were intercalated into the AS1411 G-quadruplex structure, forming a conjugate able to induce the specific accumulation of TMPyP4 into target cells and a consequent specific photodynamic therapy (PDT) following light irradiation.

Given the high aptamer versatility and the great advances in aptamer chemistry, various strategies for aptamer-drugs covalent linkage (Figure 2c) have also been proposed and can be applied to a wide range of chemicals, preserving the cost-effectiveness and often ensuring a controlled drug release.

For example, DOX was covalently liked to the anti-PTK-7 sgc8c aptamer modified with a thiol group through an acid-labile hydrazone linker that permits the release of the chemotherapeutic agent after internalization inside the acidic endosomal environment [27]. The study demonstrated that sgc8c-DOX conjugate preserved aptamer binding ability and combined the high specificity of targeting with a potency similar to the unconjugated DOX. Similarly, a pH-sensitive covalent linkage has been used to conjugate a novel dimeric anti-PSMA DNA aptamer complex (DAC) to DOX for the selective targeting of PSMA-positive cancer cells [28]. In a recent paper, Porciani et al. [29] used an RNA aptamer for transferrin receptor (TfR) to perform selective co-delivery in pancreatic tumor cells of DOX and of an inhibitor of a cell-survival factor, the nuclear factor κB (NF-κB) decoy oligonucleotide. The RNA aptamer was conjugated to the NF-κB DNA decoy by DNA self-assembly between two complementary single-stranded DNA sequences that also accommodate multiple DOX molecules. Authors demonstrated that the aptamer mediates efficient selective co-delivery that significantly increased DOX cytotoxicity toward targeted pancreatic tumor cells owing to selective inhibition of constitutive NF-κB activity. This inhibition ultimately enhanced DOX-mediated apoptotic effects.

Aptamer-DOX conjugates not only demonstrated anticancer potency similar to unconjugated DOX, but also exhibited reduced cardiotoxicity and a general limited toxicity toward non-target cells both in vitro and in vivo.

As discussed in the following sections, several aptamer-based approaches, involving the use of nanoparticles, have been proposed for drug specific delivery as an alternative to direct covalent or non-covalent conjugation.

### 2.2. Aptamer-Oligonucleotide Therapeutic Conjugates

The RNA interference (RNAi) process is a powerful tool that offers the possibility to silence the expression of specific therapeutic genes, raising the hope for a more precise medicine. However, the lack of safe and effective methods for oligonucleotide specific delivery hampers the successful translation to the clinic. To this purpose, aptamer-siRNA or miRNA conjugates (AsiCs or AmiCs) were revealed to be suitable molecules to efficiently deliver functional RNAi selectively into cells that express the cell surface receptor recognized by the aptamer. To this end, several aptamer-based approaches have been developed, allowing the targeted delivery of oligonucleotide therapeutics, including siRNAs, miRNAs, antimiRs, and antisense oligonucleotides.

**Figure 3:**
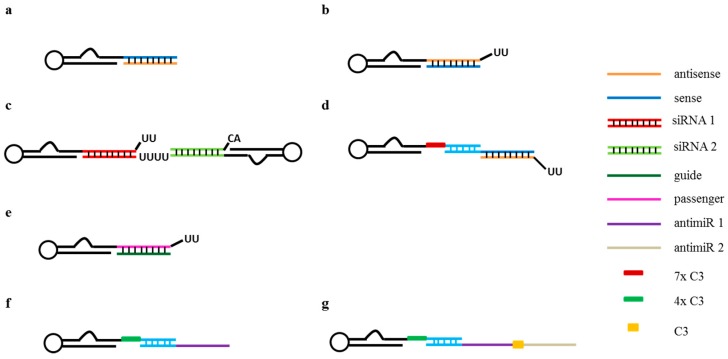
Schematic representation of aptamer-oligonucleotide conjugates. (**a**) Aptamer-siRNA chimera in which the aptamer is covalently linked to the sense strand and then the antisense is annealed; (**b**) Aptamer-siRNA chimera in which the aptamer is covalently linked to the antisense strand and then the sense is annealed. A 2-nt UU overhang at the 3’ end of the antisense strand is introduced; (**c**) Bivalent molecule in which two siRNAs are fused between two aptamers; (**d**) Stick-based approach in which the 3’ end of the aptamer is elongated with a 16-nucleotide GC-rich sequence complementary to a tail added to the siRNA; (**e**) Aptamer-miRNA conjugate in which the aptamer is covalently linked to the passenger strand and then the guide is annealed. A 2-nt UU overhang at the 3’ end of the passenger strand is introduced; (**f**) Aptamer-antimiR stick-based conjugate. The 3’ end of the aptamer is elongated with a 17-nucleotide “stick” sequence complementary to a tail added to the 3’ end of the antimiR; (**g**) Aptamer-antimiR stick-based bimodular conjugate. The aptamer is elongated with a 17-nucleotide “stick” sequence complementary to a tail added to a bimodular oligonucleotide in which the sequence of two antimiRs are spaced by a three-carbon linker.

The first AsiC system was described in 2006 by Mcnamara et al. [30] that developed completely RNA-based chimeras in which the RNA aptamer against human PSMA, A10, was covalently linked to therapeutic siRNAs targeting polo-like kinase 1 (PLK1) and BCL2, two survival genes that are overexpressed in many human cancers (Figure 3a). Generated AsiCs were revealed to bind to PSMA positive (PSMA+) cells, deliver the cytotoxic siRNAs to the RNAi machinery of target cells and induce apoptosis in vitro and in vivo in a *xenograft* mouse model of PSMA+ prostate cancer. In subsequent studies, the chimera has been modified to increase the silencing potency and the in vivo stability following intravenous injection (Figure 3b) [31]. Further, bivalent molecules, containing two anti-PSMA aptamers were generated and different designs, also permitting the contemporary delivery of multiple therapeutic siRNAs/shRNAs, were developed [32].

The signal transducer and activator of transcription 3 (STAT3) is a key downstream effector oncogene activated in several cancer types proven to be a promising target for cancer treatment [33]. Kortylewski and colleagues developed an aptamer-siRNA conjugate to selectively target the toll-like receptor 9 (TLR9) expressing immune cells of the tumor microenvironment. Authors conjugated a TLR9 agonist aptamer (CpG 1668) to a siRNA against STAT3. Although the uptake of the chimera was revealed to be not dependent on the presence of receptor, the TLR9 was essential for the functional silencing of STAT3, resulting to be effective in vivo in antitumor immune activation [34]. The same siRNA was then conjugated to the CTLA4apt [35], an aptamer raised against the cytotoxic T lymphocyte–associated antigen 4 (CTLA4). The CTLA4apt-STAT3 siRNA AsiC induced silencing of Stat3 in both CD8+ and CD4+ T cells led to reduced tumor growth in four different murine tumor models and human T cell lymphoma [36]. Further, Rossi and colleagues also described an RNA-based conjugate to treat B-cell expressing tumors. To this end, authors produced an antagonizing aptamer to target the B-cell-activating factor (BAFF) receptor with nanomolar affinity. The anti-BAFF-R aptamer was linked to a siRNA directed against STAT3, by either covalent or non-covalent conjugation. The resulting AsiC blocked ligand-mediated processes, was internalized and processed selectively in BAFF receptor expressing cells, ultimately leading to the down-regulation of STAT3 expression, proliferation and survival [37]. Other studies proved the efficacy of the aptamer against nucleolin (AS1411), a receptor over-expressed on the surface of several cancer cells and able to be internalized to the nucleus, [38] for the delivery of antisense oligonucleotides to cancer cells in vivo [39,40].

Selective delivery with an aptamer targeting the tumor-associated antigen EpCAM, that is highly expressed in epithelial cancers and their tumor-initiating cells (TIC), has been recently developed as a flexible platform for targeted therapy. To this end, the EpCAM aptamer was linked to the PLK1-specific siRNA sense strand and annealed to the antisense strand (EpCAM-AsiC), showing that the conjugate selectively induced gene silencing in the target EpCAM expressing epithelial breast cancer cells and their stem cells, while sparing normal epithelial cells and surrounding stroma. The AsiC was specifically internalized and inhibited target gene expression in EpCAM positive cancer cells and in human cancer biopsy tissues. In a very aggressive triple-negative breast cancer *xenograft* model, the EpCAM-AsiC treatment caused complete tumor regression [41]. Further, anti EpCAM aptamer-mediated survivin silencing has been explored to sensitize breast cancer stem cells to Doxorubicin [42].

From their first description, the number of aptamer-siRNA conjugates developed as cancer therapeutics has rapidly expanded [43] and more recently multi-targeting approaches have been described to further enhance the final therapeutic efficacy. For example, Liu et al. [44] generated a novel aptamer-siRNA chimera containing a bivalent anti-PSMA aptamer and two siRNAs targeting epidermal growth factor receptor (EGFR) and survivin fused between the two aptamers (Figure 3c). The conjugate was able to inhibit both EGFR and survivin expression and strongly induce apoptosis in vitro and in vivo.

Aptamer-siRNA chimeras were successfully developed also for the treatment of HIV infections [45]. Zhou et al. covalently conjugated a neutralizing aptamer directed against the HIV glycoprotein (gp120) to a siRNA targeting viral genes, which drive replication. This is the first example of a dual-function AsiC, since both the aptamer and the siRNA showed a therapeutic activity [46]. Further, the same research group identified a new anti-gp120 aptamer and described a “stick approach” for aptamer-siRNA conjugation (Figure 3d). This approach consists of the addition of a poly-carbon linker at the 3’-end of the aptamer, followed by a 16-nucleotide GC-rich sequence complementary to a tail added to the siRNA [47]. The generated conjugate was revealed to be effective in vivo in mouse models of HIV infections [48]. An optimization of this stick AsiC for in vivo development was further reported [49].

In addition, aptamer-siRNA chimeras have been proposed for cancer immunotherapy or for the treatment of immunological disorders, such as Stevens–Johnson syndrome, toxic epidermal necrolysis and graft-versus-host disease. For example, the A10 anti-PSMA aptamer was conjugated to siRNAs directed against genes implicated in the nonsense-mediated mRNA decay pathway (NMD) for cancer immunotherapy. Generated molecules were revealed to be effective in vivo in an immune-competent mouse model of prostate cancer [50]. Production of granulysin by cytotoxic T lymphocytes has been implicated as a major cause of several immunological disorders. An anti CD8 aptamer was conjugated (via a “sticky-bridge”) to a siRNA targeting the granulysin gene. AsiC was demonstrated to inhibit pathologic T-cell responses in several in vitro models of Stevens–Johnson syndrome/toxic epidermal necrolysis and graft-versus-host disease [51].

Besides aptamer-siRNA conjugates, miRNAs/antimiRs delivery has been addressed. In a first study, Dai and colleagues covalently linked an anti-Mucin aptamer to miR-29b and tested this AmiC in vitro on ovarian cancer cells. Since Mucin receptor is over-expressed in many human cancers, this approach has the potential to be effective for the treatment of different tumors. However, this study lacked in vivo experiments [52]. Recently, Esposito et al. demonstrated the in vivo effectiveness of an RNA aptamer-targeted delivery of a miRNA for the treatment of lung cancer (Figure 3e). This is a dual-function AmiC in which an RNA aptamer (GL21.T), directed against the RTK Axl, was covalently linked to miRNA let-7g. The conjugate was revealed to target tumor cells and reduce the tumor mass following systemic administration in a *xenograft* mouse model of human lung cancer [53]. The same aptamer was also conjugated to miR-212 to specifically restore TRAIL-mediated cytotoxicity in NSCLC cells [54]. On the other end, Catuogno et al. gave the first description of an aptamer-based system for the delivery of therapeutic single strand antimiRs. The GL21.T anti-Axl RNA aptamer was conjugated via a stick-bridge to the antimiR-222 (Figure 3f). The dual-function chimera was demonstrated to be effective both in vitro and in vivo in *xenograft* mouse models of human glioma. Moreover, authors developed a multifunctional chimera by conjugating, through the same stick-based approach, the GL21.T aptamer to a single strand RNA oligonucleotide containing the sequence of both antimiR-222 and antimiR-10b (Figure 3g). The multimodular conjugate was revealed to have an improved effectiveness in vitro [55]. Furthermore, miRNA and antimiR delivery has been integrated to develop a combined therapeutic approach to target glioblastoma stem-like cells (GSCs) [56]. The authors used two aptamers that bind to, and inhibit the receptor tyrosine kinases, Axl and PDGFRβ as carriers of miR-137 and antimiR-10b. They found that miR-137 and antimiR-10b synergize with the receptor inhibitory function of aptamers, effectively preventing GSC expansion. Of note, the generated conjugates are transported through an in vitro blood brain barrier (BBB) model, showing a great potential for glioma treatment.

All these studies highlight the big flexibility of aptamer-based systems for the development of oligonucleotide therapeutics for the treatment of several human diseases. Aptamer–oligonucleotide conjugates with different designs and chemical modifications, aimed to improve their pharmacodynamics and pharmacokinetics, were demonstrated to effectively reach target tissues both in vitro and in vivo and, very interestingly, to cross the BBB in vitro.

## 3. Aptamer-Nanoparticles Systems

Even if the direct aptamer-secondary reagent conjugation continues to be successfully used as delivery strategy, the combination of several kinds of nanomaterials, including gold nanoparticles (AuNPs), iron oxide nanoparticles, liposomes, block polymeric nanoparticles, carbon nanotubes, Quantum Dots (QDs) serum albumin nanoparticles and dendrimers, with cell-specific aptamers has been currently proposed and offers several advantages for targeted delivery.

Indeed, nanoparticles possess a relatively large surface area, thus allowing the incorporation of multiple targeting ligands or secondary therapeutic reagents. In addition, they have a broad and versatile chemistry that permits the development of a wide range of strategies to promote the controlled release of therapeutics.

### 3.1. Gold Nanoparticles

In the last few years, much attention has been focused on Gold nanoparticles that possess many favorable properties, including non-toxicity, biocompatibility, high stability and small dimension, allowing a wide body penetration, and the presence of functionalizable surfaces that easily permit the linkage of targeting aptamers.

**Figure 4:**
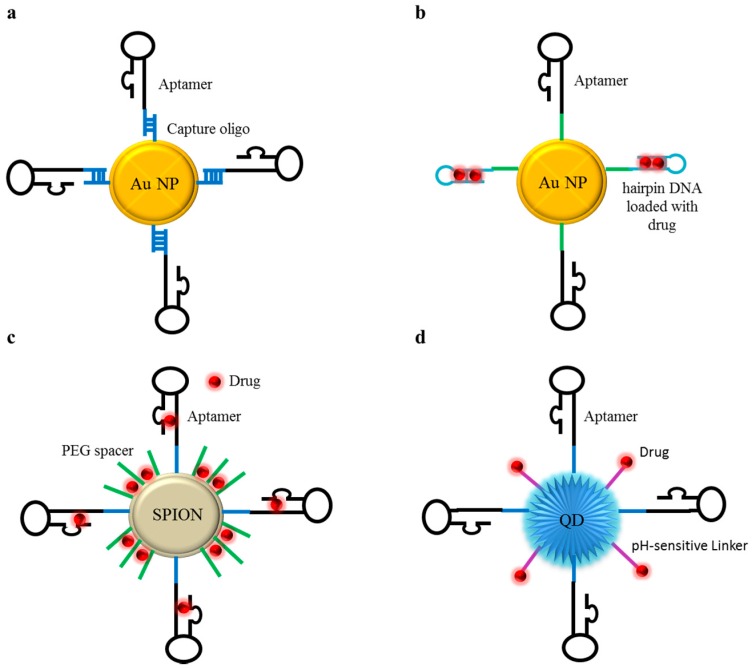
Schematic examples of aptamer- inorganic nanoparticles conjugates. (**a**) Scheme of aptamer-functionalized gold nanopaticles (AuNPs) obtained through the hybridization between short DNA capture oligos on the nanoparticle surface and complementary sequences appended to the aptamer; (**b**) Example of an AuNP-based smart drug carrier obtained by assembling on the NP surface an aptamer and a d(CGATCG)-rich hairpin DNA (hpDNA) in which doxorubicin (DOX) is intercalated; (**c**) Example of aptamer-functionalized SPIONs for targeted chemotherapy. The aptamer is covalently conjugated onto the surface of a SPION coated with a Polyethylene Glycol (PEG) spacer. DOX is loaded both by intercalation in the aptamer and by charge interactions with SPIONs; (**d**) Scheme of quantum dots (QDs) functionalized with aptamers and drugs. QDs are covalently linked to aptamers and by a pH-sensitive bond to drug molecules (i.e., DOX).

A simple non-covalent functionalization approach has been proposed by Li et al. [57] that conjugated a novel anti-EGFR RNA aptamer to AuNPs via a facile hybridization between short DNA capture sequences on the nanoparticle surface and complementary sequences appended to the 5’-end of the aptamer (Figure 4a). They showed that the aptamer specifically and quantitatively deliver the gold nanoparticles to EGFR-positive cells.

In many studies, aptamer-functionalized AuNPs have been developed for targeted therapy combining the high specificity given by the aptamer with a strong therapeutic effect. In this regard, one important feature of AuNPs is that they may be used as photothermal therapy (PTT) agents. Indeed, upon irradiation with light to a suitable wavelength, they become heat sources, thus facilitating PTT or providing a light-responsive drug delivery platform that allows a remote control (by light illumination) of the drug release.

For example, Luo et al. [58] devised a smart drug carrier by assembling onto the surface of AuNPs the sgs8c DNA an aptamer and a hairpin DNA (hpDNA) containing the repeated d(CGATCG) for DOX loading (Figure 4b). Oligonucleotide attachment on nanoparticles was obtained through standard gold-thiol chemistry. The study demonstrated a selective binding capability of the conjugates and, most importantly, showed that the exposure to plasmon-resonant light (532 nm), induced a marked release of DOX, resulting in an enhanced and specific anti-tumor efficacy. A similar controlled release approach has been proposed by Yang et al [59]. They used mesoporous silica-coated gold nanorods that were functionalized with AS1411 aptamers by the annealing of complementary strands. Upon exposure to near-infrared (NIR) light, the increased temperature of Au nanorods, due to photothermal effect, resulted in the dehybrization of the duplex DNA that anchored the aptamer on the surface, allowing the release of entrapped drugs as Doxorubicin.

Gold nanorods were also functionalized with sgc8 DNA aptamer for targeted combined PDT and PTT by Tan group [60]. The aptamer was modified to generate a switch probe (ASP) that was conjugated with the 5’-end to AuNPs by thiol chemistry and with 3’-end to chlorin e6 (Ce6), a photosensitizing molecule. Upon binding to the target cells, ASP changed its conformation, moving Ce6 away from the gold surface. Consequently, there was the production of singlet oxygens inducing PDT following light irradiation (812 nm). Since radiation also resulted in AuNP photothermal effects, these conjugates combined PDT to PTT, getting a greater effect than single therapies.

In a following study, pH-sensitive anti-CD30 aptamer- hollow gold nanosphere (HAuNS) complexes loaded with DOX (Apt-HAuNS-Dox) have been developed [61]. It has been demonstrated that Apt-HAuNS-Dox was internalized exclusively into the targeted lymphoma tumor cells releasing 80% of the drug within 2 h at the acid pH of cell lysosomes, thus selectively killing target cells without any effect on off-target cells grown in the same culture.

A nice co-drug delivery was proposed by Shiao et al. [62]. In their study, aptamer-based Au-NPs have been used to co-deliver photosensitizer TMPyP4 and Doxorubicin. The anti-nucleolin AS1411 aptamers were assembled on the nanoparticle surface by a dsDNA annealing and both TMPyP4 and DOX were loaded by simple physical intercalation into the aptamer sequences. Light exposition determined a specific TMPyP4-depedent photo damage and a simultaneous release of Doxorubicin, thus increasing the final therapeutic effect.

More recently, novel AuNP-aptamer complexes have been designed for the anthracycline drug Daunorubicin (Dau) pH-sensitive delivery to leukemia cells [63,64]. In a first study [63], anti-PTK-7 sgc8c aptamers were attached to AuNPs through electrostatic interaction and Dau was loaded both by electrostatically adsorption onto the nanoparticle surface and intercalation to polyvalent aptamers. The complex showed efficient and selective binding and cytotoxicity in vitro. The same strategy was then further developed [64] to obtain a double targeting system by using blocks of the polyvalent aptamer system containing the anti-PTK-7 sgc8c and the anti-nuleolin AS1411 aptamers. In addition to its selectivity, the possibility to effectively reverse the functional effect of the complex using an antisense of the polyvalent aptamers was demonstrated. AuNP-based dual targeting was also obtained by designing a novel nano-platform containing cyclic Arg-Gly-Asp (cRGD) that is specific for αvβ3integrins, and the AS1411 aptamer [65]. The complex was further functionalized with near infrared fluorescence dye or DOX, to produce a dual-targeting NIR fluorescent probe or pro-drug with a favorable tumor-targeting both in vitro and in vivo.

In addition to small chemotherapeutic agents, the use of AuNP-aptamer systems for biologically functional peptides delivery has been addressed. Ryou et al. [66] proposed a gold nanoparticle-DNA aptamer-based system in which purified His-tagged proteins were loaded onto AuNPs conjugated with a His-tag aptamer (AuNP-His-Apt) by simple mixing and incubation. The system was successfully used to deliver several proteins into a variety of cell types in vitro. In addition, the authors found that AuNP-Apt loaded with the apoptosis-inducing Bcl-2-like protein 11 (BIM) were therapeutically effective in vivo. This strategy has been further applied to drive to lung cancer cells the functional domain of Discoidin domain receptor 2 (DDR2), a collagen-induced receptor tyrosine kinase that is emerged as a novel therapeutic target in lung cancer. It has been shown that the AuNP-Apt-based peptide complex efficiently inhibited DDR2 activation, decreasing cell proliferation and invasion [67].

In a recent study, AuNPs have been combined to graphene oxide (GO) to generate a novel anti-MUC-1 aptamer-gold nanoparticle-hybridized GO nanocomposite (Apt-AuNP-GO) able to induce targeted cancer cell PTT at ultralow concentrations [68].

Even if all the discussed studies provide encouraging results on the potential therapeutic applicability of aptamer-AuNPs complexes, it is important to note that only few of these include in vivo experiments addressing important issues i.e. complex accumulation and toxicity. In this regard, Dam et al. [69] published a promising study with an anisotropic Au nanoconstruct composed of a gold nanostar core functionalized with an AS1411 anti-nucleolin aptamer. The authors found no signs of acute toxicity at the highest dose tested (48 mg/kg) and a tumor specific accumulation five times higher in invasive breast cancer tumors compared to fibrosarcoma tumors.

### 3.2. Magnetic and Superparamagnetic Iron Oxide Nanoparticles

Superparamagnetic iron oxide nanoparticles (SPIONs) are one of the most interesting magnetic NPs especially for their suitability as theranostic agents. In addition to being endowed with diagnostic capabilities in the magnetic resonance imaging (MRI) approach, they have attracted growing interest since their surface may be easily modified for the development of targeted nanoparticle platforms.

By using thermally cross-linked SPION (TCL-SPION), Wang et al. [70] developed novel bio-conjugates (TCL-SPION-Apt) for combined prostate cancer imaging and therapy. The anti-PSMA A10 aptamer was used as a targeting moiety; the aptamer was modified with C18-amine at the 3′ end to bind to TCL-SPION coated with an anti-biofouling polymer derived from carboxylic acid- polyethylene glycol (PEG). DOX was loaded by intercalation in the aptamer and by charge interactions with TCL-SPION (Figure 4c). The study demonstrated that TCL-SPION-Apt conjugate functioned as an MRI contrast agent, permitting the detection of PSMA-positive prostate cancer cells with high sensitivity, and selectively inducing Doxorubicin cytotoxicity into target cells in vitro. TCL-SPION have been also used to design a dual-aptamer complex able to target both PSMA positive and negative prostate cancer cells [71]. To this end both A10 (targeting PSMA positive cells) and anti-DUP-1 peptide (targeting PSMA negative cells) aptamers were covalently immobilized on the surface of TCL-SPION, then loaded with DOX. Bi-specific cell uptake and drug delivery were verified in vitro. However, no in vivo data were reported in these studies.

Subsequently, a SPION-based approach has been extended to other cancer cells by generating a tertiary complex, combining SPIONs with the anti-MUC-1 5TR1 aptamer and the anthracycline drug Epirubicin [72]. The study demonstrated that the conjugate specifically bound and internalized in murine colon carcinoma target cells, specifically reducing cell viability in vitro. Of note, the complex was successfully used for colon cancer imaging and therapy in vivo.

Recently, SPIONs were also combined with gold materials to generate a self-therapeutic device for colon cancer [73]. Gold-coated superparamagnetic iron oxide nanoparticles (Au@SPIONs) were conjugated to a thiol modified MUC-1 aptamer used as a targeting moiety. The generated complex functioned as a theranostic agent providing a MR imaging and photothermal therapy tool. A DNA-RNA hybrid aptamer targeting PSMA was instead used to functionalize, via biotin-streptavidin coupling, SPIONPs loaded with DOX. The resulting SPIO-Apt-Dox was effectively employed as a specific delivery platform for prostate cancer therapy [74].

In addition to SPIONs, other magnetic nanoparticles have been explored. For example, a theranostic nanoprobe has been developed by combining cancer targeting of a DNA-based EpCAM aptamer with the imaging capability of magnetic iron oxide nanoparticles modified with carboxymethyl cellulose [75]. The proof-of-concept study demonstrated good MR imaging, drug delivery and therapeutic efficacy in vitro. Magnetic fluorescent (MF) nanoparticles were instead used to develop a multifunctional cancer-targeting theranostic system (MFAS) containing DNA aptamer (AS1411) and miRNA molecular beacon (MB) complementary to miR-221 [76]. The system was generated by covalently conjugating 5′ amine-modified AS1411 aptamer or amine-modified miR-221 MB to mesoporous silica nanoparticles (MSNs) coated with a PEG–COOH spacer. After internalization into nucleolin positive target cells, the miR-221 MB was released from the MFAS and recognized the complementary miR-221, allowing the simultaneous imaging of it and inhibiting its oncogenic function.

Mentioned studies demonstrated that aptamer-SPION conjugates are able to specifically target the tumor, allowing the targeted delivery of anticancer drugs both in vitro and in vivo and efficient in vivo imaging for cancer diagnosis.

### 3.3. Quantum Dots

Quantum dots are semiconductor nanocrystals with unique optical properties. So far, they have been widely combined to aptamers or other targeting ligands to generate extraordinary molecular biosensors for real-time tracking and imaging [77]. Nevertheless, recent papers have described their use for targeted drug delivery.

Savla et al. [78] developed a pH-sensitive quantum dot-Mucin 1 aptamer-DOX conjugate (QD-MUC1-DOX) for ovarian cancer chemotherapy. A pH-responsive hydrazone bond was used to attach DOX to QDs previously covalently linked to the MUC-1 DNA aptamer (Figure 4d). The data obtained by confocal microscopy and in vivo imaging showed that QD-MUC1-DOX conjugate preferentially accumulated in tumors, inducing higher cytotoxic than free DOX in multidrug-resistant cancer cells.

A nice traceable and dual-targeted drug delivery system was instead developed by Zhang et al. [79] by using DNA-hybrid-capped mesoporous silica-coated quantum dots (MSQDs). MSQDs were loaded with Doxorubicin and then conjugated via annealing with a DNA hybrid containing the anti-nucleolin AS1411 aptamer extended at the 3’ with the antimiR-21. In this way, a DNA gate that prevents DOX release and is regulated by miR-21 is formed. The authors demonstrated that only when the complex, by recognizing nucleolin, entered target cells overexpressing miR-21, the antimiR hybridized with the miR and unlocked the gate, releasing the DOX and simultaneously inhibiting miR-21. This mechanism further enhanced the efficacy of the chemotherapy.

A different stimuli-responsive drug delivery system involving graphene quantum dots (GQDs) has been then proposed by Zheng and co-workers [80]. They developed a smart drug nanocarrier based on fluorescence resonance energy transfer (FRET). GQDs were capped onto fluorescent mesoporous silica nanoparticles (FMSNs) conjugated to an ATP aptamer (functionalization moiety) and the anti-nucleolin AS1411 aptamer (targeting moiety) and loaded with DOX by intercalation. The system was designed so that at low ATP level, the fluorescence of FMSNs and the DOX release remained in an “off” state. When AS1411 aptamer-mediated internalization occurred, the ATP-rich cytoplasm context induced a conformational switch of the ATP aptamer that caused the shedding of the GQDs from the nanocarriers with a consequent release of the loaded drugs and simultaneous activation of FMSNs fluorescence. This allowed a FRET-based real-time monitoring of the drug release in vitro.

In another study, novel daunorubicin-loaded NIR CuInS_2_ quantum dots were developed by using an anti-MUC-1 aptamer as the targeting moiety [81]. The complex (DNR-MUC1-QDs) contained the MUC-1 aptamer linked on the QD surface through a GC reach complementary duplex in which daunorubicin was intercalated. The study showed that the developed system effectively delivered the drug to target prostate cancer cells in vitro and enhanced daunorubicin sensing by the change of photoluminescence intensity of CuInS_2_ that simultaneously allowed the imaging of the cells.

The theranostic capabilities of nutlin-3a loaded poly (lactide-co-glycolide) nanoparticles, functionalized with a EpCAM aptamer as the targeting ligand and quantum dots as the imaging agent, has also been recently explored [82]. This multifunctional structure demonstrated promising therapeutic potential and served as a bio-imaging tool in EpCAM expressing cancer systems in vitro.

All together, these studies represent promising examples of theranostic agents for simultaneous cancer therapy and bioimaging, however, as for other NPs, their applicability in vivo has not yet been rigorously investigated, and further steps are still necessary for their validation.

### 3.4. Carbon Nanomaterials

**Figure 5:**
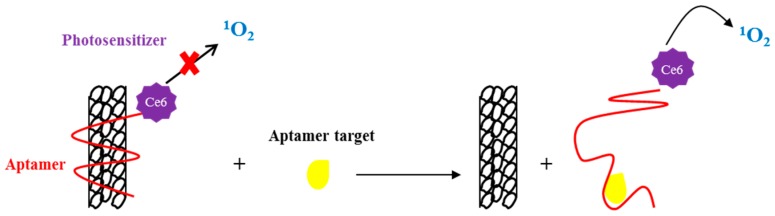
Schematic representation of aptamer-photosensitizer- single-walled carbon nanotubes (SWNT) conjugate. The aptamer, linked to the photosensitizer, is wrapped onto the SWNTs surface and switches off the singlet oxygen generation (SOG). In the presence of the aptamer target, the interaction between the aptamer and SWNTs is inhibited and the SOG is restored.

Among carbon nanomaterials, single-walled carbon nanotubes (SWNTs) represent a class of promising tools for targeted drug delivery, due to their advantageous properties: (1) easy internalization into target cells; (2) high capacity of drug loading and a wide surface area to attach targeting molecules; (3) interesting optical and photoacoustic properties [83]. Zhu and coworkers generated carbon nanotubes functionalized with an aptamer on their surface, to control drug release using photodynamic therapy. A photosensitizer (Ce6) was covalently attached to one end of the aptamer. In the presence of the aptamer target, the binding allowed the aptamer to fall off the SWNT surface, resulting in a restoration of singlet oxygen generation (SOG) for photodynamic therapy. Thus, the aptamer reversibly controlled the photosensitizer’s activity (Figure 5) [84]. In a recent study, SWNTs were used as drug carriers for NIR light-controlled cancer cell delivery and killing. SWNTs were conjugated to sgc8 aptamers that were caged by the hybridization to complementary DNA strands. Upon NIR laser exposure, the photothermal heating induced the DNA strand dehybridization, exposing the aptamers that were able to specifically recognize target cells, guiding the delivery of secondary reagents (DOX) encapsulated into the NPs. The killing activity was specifically induced by the presence of the NIR light, indicating that PEGylated SWNTs were not toxic for cells. Reduction of cell viability was evaluated by an MTT (3-(4,5-dimethylthiazol-2-yl)-2,5-diphenyltetrazolium bromide) assay on human T lymphoblast [85].

Graphene oxide is another carbon nanomaterial that has been conjugated with aptamers for the targeted delivery of drugs. For example, Lu et al. developed cell specific aptamer-functionalized graphene oxide nanoparticles to deliver decitabine specifically to A549 non-small cell lung cancer (NSCLC) cells. Graphene-based conjugates were revealed to be more effective than the free drug [86].

Mentioned studies have demonstrated the great potential of carbon nanomaterials in targeted therapy. However, it is necessary to carefully adjust the concentration, the physical form and the degree of functionalization of these compounds to reduce their toxicity. Moreover, their effectiveness in vivo has yet to be proven, since to date only in vitro results have been obtained [87,88].

### 3.5. Liposomes

**Figure 6:**
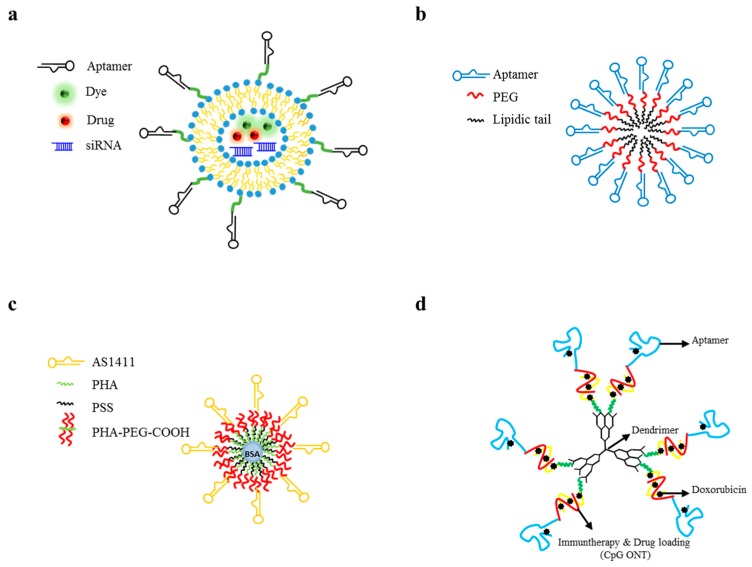
Examples of aptamer-functionalized liposome, micelle, serum albumin or dendrimer nanoparticles. (**a**) Scheme of aptamer-functionalized liposomes. Aptamer is covalently conjugated to the surface of the liposome, whose core may be encapsulated with different secondary reagents (dyes, drugs, siRNAs); (**b**) Schematic representation of PEGylated aptamer-micelle formation; (**c**) Schematic representation of aptamer-based bovine serum albumin (BSA) nanoparticles coated with poly(allylamine hydrochloride) (PAH)/sodium poly(4-styrene sulfonate) (PSS) multilayers; (**d**) Aptamer-dendrimer bioconjugates for targeted chemo-immunotherapy. Aptamer is conjugated to Poly(amidoamine) (PAMAM) dendrimers through the hybridization between complementary CpG-rich oligonucleotides appended to the aptamer and on the NPs. The generated duplexes function as a loading site for DOX and as an immune-stimulating agent.

Liposomes are spherical phospholipid-based structures that have been extensively applied as drug-delivery systems. Their specific structural organization allows both the encapsulation of hydrophilic therapeutics into their aqueous core and the loading of hydrophobic molecules inside their lipid bilayer membrane. To date, several liposome-based systems have been approved for clinical use and are in clinical trials [89]. Given the fundamental importance of therapeutic selective delivery, there are a growing number of papers exploring liposome combination to molecular recognition moieties, including aptamers (Figure 6a).

A first aptamer-liposome delivery system was developed by Cao et al. [90]. In their approach, the anti-nucleolin aptamer sequence was modified at the 3’-end with a 12-thymine spacer followed by a cholesterol tag for the immobilization on a PEGylated liposome hydrophobic surface, and the hydrophilic dye calcein (to monitor internalization) or the chemotherapeutic drug cisplatin (to induce anti-proliferation activity) were encapsulated into the liposome core. Notably, in addition to demonstrating the specific targeting of breast cancer cells overexpressing nucleolin, the authors designed a complementary DNA able to disrupt the aptamer-structure reversing the drug delivery. The anti nucleolin aptamer was also used to functionalize liposomes containing doxorubicin as a payload [91]. Recently, the same group systematically investigated the effects of spacer length and PEG composition on aptamer-liposomes loaded with DOX. They found that a spacer length able to assure the full exposition of the aptamer on the liposomal surface, is critical for the best targeting effect, regardless of its composition [92].

Tan group described a different aptamer–liposome conjugate, also achieving cancer cell specific targeting [93]. The sgc8 DNA aptamer was covalently conjugated by maleimide chemistry to the surface of liposomes and a PEG polymer-coating was introduced to increase complex stability in serum. The liposome core was loaded with FITC-Dextran. The study demonstrated an efficient and specific targeting of cancer cells in vitro. Similarly, more recently, Alshaer and co-workers [94] conjugated, by thiol-maleimide click reaction, an anti-CD44 RNA aptamer to the surface of PEGylated liposomes, achieving a higher sensitivity and selectivity compared to the blank liposomes.

Another approach was proposed by Baek et al. [95] that designed a novel Doxorubicin-encapsulating liposome conjugated with the anti PSMA A9 aptamer. The aptamer–liposome complex (called aptamosome) was obtained through a post-insertion method based on the insertion into liposome of aptamer-conjugated micelles. DOX-encapsulating aptamosomes showed enhanced prostate cancer cell (PSMA positive) binding and uptake in vitro and, most importantly, was selectively retained in tumor tissue in vivo leading to reduced tumor size in *xenograft* mice. Based on these papers, so far, several other aptamers have been employed for DOX-encapsulating liposomes’ selective delivery [96,97].

Aptamer–liposome conjugates have been also proposed for siRNA selective delivery. Willer et al. [98] selected a new serum-stabilized aptamer, able to recognize the human transferrin receptor, and used this oligonucleotide for the delivery of nucleic acid-lipid particles (SNALPs) containing siRNA cargoes. A 5’ thiol-modified aptamer was used for the covalent conjugation to the liposomes. As expected, the functionalized SNALPs showed enhanced uptake and target gene knockdown in cultured target cells without inducing any adverse cellular effects.

In addition, more recently, Stuart et al. proposed a new aptamer-functionalized liposomal formulation for the specific delivery to prostate cancer cells of the zinc chelator, *N*,*N*,*N*’,*N*’-tetrakis(2-pyridylmethyl)-ethylenediamine (TPEN). TPEN action results in unbalancing oxygen species, inducing cell death. The authors showed that the aptamer-targeted liposome- TPEN complexes specifically targeted prostate cancer cell both in vitro and in vivo [99]. Of note, a recent paper reported a multifunctional liposomal system for the simultaneous delivery of DOX, heat, and a bubble-generating agent (ammonium bicarbonate, ABC) [100]. The anti-MUC-1 aptamer was conjugated on the surface of liposomes encapsulated with gold nanocages, ABC and DOX. Upon NIR light irradiation, the gold NPs generated localized heat, inducing the decomposition of ABC, which in turn triggered DOX release by forming permeable defects in the lipid bilayer. In addition, the targeting aptamer was modified to function as an activatable molecular beacon in order to track the internalization of the complexes and activate photothermal effect when it reached the maximum.

Of note, in vivo studies in nude mice demonstrated that liposomes’ aptamer-conjugation increases the accumulation of DOX in tumor tissues relative to free DOX or passively targeted plain liposomes, inhibiting tumor growth and reducing systemic side effects, including cardiotoxicity [101].

### 3.6. Block Polymeric Nanoparticles

Poly(lactide-co-glycolic acid) (PLGA) is a biocompatible and biodegradable polymer approved by the FDA. Different studies demonstrated the effectiveness and safety of drug delivery systems based on PLGA nanoparticles [102]. PLGA nanoparticles are usually functionalized with polyethylene glycol since PEGylated polymeric nanoparticles show significantly reduced systemic clearance compared with nanoparticles without PEG [103,104]. Farokhzad and colleagues developed paclitaxel and docetaxel (dtxl)-encapsulated PLGA nanoparticles functionalized with PEG and conjugated with the anti-PSMA A10 aptamer on the surface, in order to increase the specificity and reduce the overall toxicity. The anti-cancer activity of this type of conjugate was proved both in vitro and in vivo. Interestingly, after systemic administration in human xenograft prostate cancer mice, the intra-tumoral concentrations of the drug was higher than that observed in the control group treated with nanoparticles not functionalized with the aptamer. Biodistributions to other organs (liver, heart, lungs, and kidneys) did not show substantial accumulation in either group and were not significantly different [105]. A10 aptamer-functionalized PLGA-PEG nanoparticles were also used as carriers to deliver platinum (IV) prodrug in prostate cancer following intravenous injection. Conjugates were demonstrated to be effective in reducing prostate tumors both in vitro and in vivo, with a significantly low dose of platinum [106,107].

Further, Gu et al. generated a triblock copolymer (TCP) consisting of PLGA, PEG, and the A10 aptamer. This TCP could be used for the self-assembly of targeted nanoparticles for prostate cancer targeting. They demonstrated rapid endocytosis of the bioconjugate by confocal microscopy in PSMA+ LNCaP prostate cancer cells. Moreover, they found that increasing aptamer density on the surface area of nanoparticles is able to increase the rate of nanoparticles uptake by tumor cells in vitro and their accumulation in liver and spleen in vivo in LNCaP xenograft mouse models of prostate cancer. Thus, following different attempts, authors established the optimal ratio to obtain the maximal cell uptake [108].

More recently polyethylene glycol-poly(lactic-co-glycolic acid)-based NPs were functionalized with AS1411 anti-nucleolin aptamer and loaded with gemcitabine [109]. Cellular uptake of the generated complex was evaluated in vitro by flow-cytometry analysis and fluorescent microscopy on A549 NSCLC nucleolin positive cells. Moreover, the cytotoxicity and the IC_50_ of the bioconjugate was evaluated in vitro in A549 nucleolin positive and Chinese hamster ovary (CHO) nucleolin negative cells. Obtained results demonstrated that the IC_50_ value on A549 for aptamer-functionalized NPs was much lower than non-functionalized NPs, whereas they were similar in CHO, indicating a good target specificity.

In addition to PLGA, a polyethylenimine (PEI)-based noncomplex that employs the EpCAM aptamer and EpCAM siRNA was developed for siRNA delivery to EpCAM positive cancer cells. Cellular uptake of the complex was evaluated in vitro by flow cytometry and fluorescent microscopy. Inhibition of cancer cell proliferation was evaluated by an MTT assay [110].

### 3.7. Polymeric Micelles

When the ratio of hydrophobic and hydrophilic components in copolymers is appropriate, they could self-assemble into micelles in an aqueous environment [111,112]. Micelles as drug carriers show very interesting properties, including high solubility and bioavailability of hydrophobic drugs in water, long-term stability in blood and slow drug release rate. Conjugation with aptamers confers high target specificity (Figure 6b).

First, PEGylated micelles, functionalized with an anti-B-cell lymphoma DNA aptamer, were developed and revealed binding selectivity in vitro as assessed by flow cytometry and internalization into target cells as evaluated by fluorescent microscopy. Aptamer–micelle conjugates showed great dynamic specificity in flow channel systems that mimic drug delivery in blood circulation, demonstrating a possible in vivo applicability [113]. Hao and colleagues successfully developed aptamer-functionalized PEG-polylactic acid micelles (APPs), containing the anti-inflammatory molecule flurbiprofen, for targeting the transferrin receptor in brain endothelial cells. In this study the authors analyzed the in vitro release profile of APPs and observed that, compared to free drug, PEG–PLA micelles controlled the release of flurbiprofen by prolonging the release period. Moreover, they observed that a good percentage of flurbiprofen was retained in micelles following FBS and BSA incubation. Accumulation of flurbiprofen in target cells, binding activity and intracellular uptake of micelle were also confirmed in vitro. However, the study is lacking in vivo data [114].

Aptamer-conjugated pH-sensitive micelles were also developed. A pH-responsive nanostructure consisting of a hyperbranched H40 polymer core and approximately 25 amphiphilic polylactide (PLA)-PEG block copolymer arms, loaded with DOX. The release rate of DOX was revealed to be much higher at a pH of 5.3 than at a pH of 7.4 in vitro. Binding, cellular uptake and specific cytotoxicity were also demonstrated in vitro. Interestingly, the in vivo biodistributions of free DOX, DOX-loaded targeted and non-targeted micelles were compared in prostate tumor models. Aptamer-mediated delivery demonstrated the best release of DOX in the tumor site [115]. Further, Chen et al. developed aptamer-functionalized micelles consisting of a pH-sensitive copolymer, D-α-tocopherol PEG 1000-block-poly-(β-amino ester) (TPGS-b-PBAE, TP) encapsulating paclitaxel. The resulting micelles were stable at pH 7 but dissolved and quickly released paclitaxel in an acidic environment (pH 5.5). These micelles were able to induce G2/M phase arrest more efficiently than free paclitaxel [116]. In a recent study, [117] a multifunctional micelle functionalized with the anti-nucleolin aptamer has been described for targeted delivery of DOX to breast tumors. The complex consisted of aptamer modified Pluronic F127 and beta-cyclodextrin-linked poly(ethylene glycol)-b-polylactide and showed enhanced DOX-loading and micelle stability. Notably, the authors demonstrated prolonged permanence in blood, improved accumulation in tumor and antitumor activity with a decreased cardiotoxicity in vivo.

Based on the discussed examples, the use of biodegradable micelles for targeted drug delivery represents a very interesting prospective for human therapy. However, many challenges have to be addressed to overcome some limitations, such as the low stability in vivo, the poor tumor penetration and cellular uptake.

### 3.8. Serum Albumin Nanoparticles

Bovine serum albumin (BSA)-based nanoparticles show several advantages as drug delivery tools. They are biodegradable and safe molecules, since they can be absorbed by the human body without producing toxic residual substances. Moreover, they are easy to prepare and show a high storage stability and a good performance of controlled release [118]. The hydrophobic core of these nanoparticles shows high affinity for hydrophobic drugs and can offer various possibilities of conjugation. Human serum albumin-bound paclitaxel nanoparticles (Abraxane), showing high effectiveness and reduced side effects, have been approved by the FDA for the treatment of metastatic breast cancer [119]. Moreover, BSA-nanoparticles conjugated with aptamers were revealed to be promising delivery tools for the clinical applications.

In an interesting example, Gao et al. generated BSA nanoparticles using a desolvation technique and on the surface the AS1411 an aptamer was covalently bound. Nanoparticles were then loaded with DOX. The resulting conjugates showed a releasing rate higher at pH 5.5 than at pH 7.4 and induced liver cancer cells death more effectively than the free drug (Figure 6c) [120].

### 3.9. Dendrimers

Dendrimers are multibranched polymers with a central inner core surrounded by layers of repeated units with an outermost layer of multivalent functional groups. Due to the large number of terminal branching units, dendrimers can be functionalized with multiple molecules through non-covalent bonds, such us hydrogen, van der Waals, and electrostatic interactions [121]. For their features, dendrimers can be used to generate innovative combined approaches with great potential as therapeutics. Moreover, they show a narrow polydispersity that allows their easy passage across the biological barriers. Recently, aptamer-dendrimer bioconjugates with high target specificity have been developed. Poly(amidoamine) (PAMAM) succinamic acid dendrimers were conjugated with oligodeoxynucleotides on the outermost layer, resulting in single-stranded oligodeoxynucleotide-conjugated dendrimers (sONT-DENs). Then, anti-PSMA aptamers were hybridized with the sONT-DENs, creating double-stranded aptamer-dONT-DEN conjugates and then DOX was intercalated (Figure 6d). Resulting bioconjugates revealed antitumor properties and target specificity in vivo in prostate tumor models [122]. In a recent paper, PAMAM-PEG-based dendrimers were conjugated to the anti-nucleolin aptamer and 5-fluorouracil (5-FU) chemotherapeutic drug, achieving 5-FU specific accumulation in target cancer cells [123]. Further, Taghdisi et al. used three aptamers (MUC-1, AS1411 and ATP aptamers) to functionalize dendrimer for the targeted delivery of Epirubicin [124]. The study demonstrated that the complex was specifically internalized into target cells and showed effective cytotoxicity in vitro and efficient inhibition of tumor growth in vivo.

In addition to drug delivery, the aptamer-conjugated dendrimer has been successfully used for miR delivery [125]. The tumor suppressor miR-34a was encapsulated into a dendrimer linked to an anti-lung cancer cells aptamer (S6), significantly improving miR cellular uptake in NSCLC cells. The generated complex hampered cell growth, migration, and invasion and induced apoptosis in vitro.

## 4. Conclusion and Future Perspectives

Since their first discovery in 1990 [126,127], aptamers have attracted a growing interest as novel targeting molecules. The possibility to easily select them against practically any target of interest is a powerful feature that has greatly spread their applicability in the therapeutic and diagnostic fields. In addition, to provide therapeutic or diagnostic reagents, because of their high specificity, low toxicity and internalizing properties, aptamers against cell surface molecules have demonstrated their versatility as effective recognition ligands for the selective delivery of various secondary reagents to diseased cells.

As discussed in this review, an increasing number of cell surface epitope targeting aptamers have been successfully and extensively used as delivery carriers by direct conjugation to therapeutic compounds. In addition, thanks to the aptamer chemical versatility, many approaches have been developed to use them for nanoparticle functionalization. Indeed, blank nanoparticle-based conjugates improve membrane permeability and increase circulation half-life, but are selectively trapped in the liver [103]. A growing number of studies has demonstrated that this may be overcame by using nucleic acid aptamers. Indeed, conjugation with aptamers can improve the efficacy and the biodistribution of molecules thanks to their high targeting specificity, reducing the accumulation in non-target tissues and thus the occurrence of off-target effects. Chemical modifications and conjugation with PEG can be easily made to increase conjugate serum stability and reduce the renal clearance. All together, the described examples underline a great potential therapeutic applicability of aptamer-based complexes. Nevertheless, important issues remain to be tackled for their rapid transition to the clinic. Even if it is well demonstrated that aptamer internalization occurs in a receptor-mediated manner, the mechanism underlining the processing, the endosomal escape and the drug release from the aptamers are still not completely understood and a deeper comprehension of these aspects may provide important information to further optimize the effectiveness of the conjugates. Most importantly, a more rigorous investigation of their applicability and toxicological aspects in vivo are required. In addition, to achieve clinical translation, large-scale production costs should be taken into account and still represent an important challenge.

These above aspects should be thus efficiently pursued in the near future in order to realize the promise of efficient and safe aptamer-based targeted therapies to be applied in the clinic routine.

## Abbreviations

The following abbreviations are used in this manuscript:

ABC	ammonium bicarbonate
AmiCs	aptamer-miRNA conjugates
APPs	aptamer-functionalized PEG-polylactic acid micelles
AsiCs	aptamer-siRNA conjugates
AuNP	gold nanoparticle
BAFF	B-cell-activating factor
BBB	Blood Brain Barrier
BSA	Bovine serum albumin
C	cytosine
CHO	Chinese hamster ovary
CTLA4	cytotoxic T lymphocyte–associated antigen 4
Dau	Daunorubicin
DDA	Drug-DNA Adduct
DDR2	Discoidin domain receptor 2
DOX	Doxorubicin
Dtxl	docetaxel
EGFR	Epidermal growth factor receptor
EpCAM	epithelial cell adhesion molecule
FDA	Food and Drug Administration
FMSNs	fluorescent mesoporous silica nanoparticles
FRET	fluorescence resonance energy transfer
G	guanine
GO	graphene oxide
GSC	glioblastoma stem-like cell
GQDs	graphene quantum dots
HAuNS	hollow gold nanosphere
MB	molecular beacon
MF	Magnetic fluorescent
MFAS	multifunctional cancer-targeting theranostic system
MRI	magnetic resonance imaging
MSNs	Mesoporous silica nanoparticles
MSQDs	mesoporous silica-coated quantum dots
MTT	3-(4,5-dimethylthiazol-2-yl)-2,5-diphenyltetrazolium bromide
MUC	Mucin
NF-κB	nuclear factor κB
NIR	near infrared
NMD	nonsense-mediated mRNA decay pathway
NPs	nanoparticles
NSCLC	non small cell lung cancer
PAMAM	Poly(amidoamine)
PDT	photodynamic therapy
PEG	polyethylene glycol
PEI	polyethylenimine
PLA	amphiphilic polylactide
PLGA	Poly(lactide-co-glycolic acid)
PLK1	polo-like kinase 1
PSMA	Prostate-Specific Membrane Antigen
PTK	protein tyrosine kinase
PTT	photothermal therapy
QD	Quantum Dot
RNAi	RNA interference
SELEX	Systematic Evolution of Ligand by EXponential enrichment
SNALPs	nucleic acid-lipid particles containing siRNA cargoes
SOG	singlet oxygen generation
sONT-DENs	single-stranded oligodeoxynucleotide-conjugated dendrimers
SPIONs	Superparamagnetic iron oxide nanoparticles
STAT3	signal transducer and activator of transcription 3
SWNTs	single-walled carbon nanotubes
TCL	thermally cross-linked
TCP	triblock copolymer
TfR	transferrin receptor
TIC	tumor-initiating cells
TLR9	toll like receptor 9
TPEN	*N*,*N*,*N’*,*N’*-tetrakis(2-pyridylmethyl)-ethylenediamine
5-FU	5-fluorouracil
